# Supplementary material for: Utility of the Ribosomal Gene 18S rRNA in the Classification of the Main House Dust Mites Involved in Hypersensitivity
Source: Int J Mol Sci. 2025 Oct 23;26(21):10308. doi: 10.3390/ijms262110308 (PMC12607703; doi:10.3390/ijms262110308)
Supplement: Supplementary file 1 [file ijms-26-10308-s001.zip › ijms-3768634-Table S1.pdf]

Material suplementario

Species Sample	Concentration of extracted DNA	Absorbance 260/280 nm	Absorbance 260/230 nm	Size bp
Dermatophagoides pteronyssinus	54.9 ng/µl	1.969	1.911	397
Dermatophagoides farinae	72.3 ng/µl	2.102	2.204	403
Tyrophagus putrescentiae	146.6 ng/µl	2.202	2.024	554
Blomia tropicalis	59.0 ng/µl	2.167	1.779	390
Lepidoglyphus destructor	81.3 ng/µl	1.919	1.660	540

Table S1: Concentration and purity of extracted genomic DNA
