# Supplementary material for: Utility of the Ribosomal Gene 18S rRNA in the Classification of the Main House Dust Mites Involved in Hypersensitivity
Source: Int J Mol Sci. 2025 Oct 23;26(21):10308. doi: 10.3390/ijms262110308 (PMC12607703; doi:10.3390/ijms262110308)
Supplement: Supplementary file 1 [file ijms-26-10308-s001.zip › ijms-3768634-Figure S1.pdf]

Figura S1

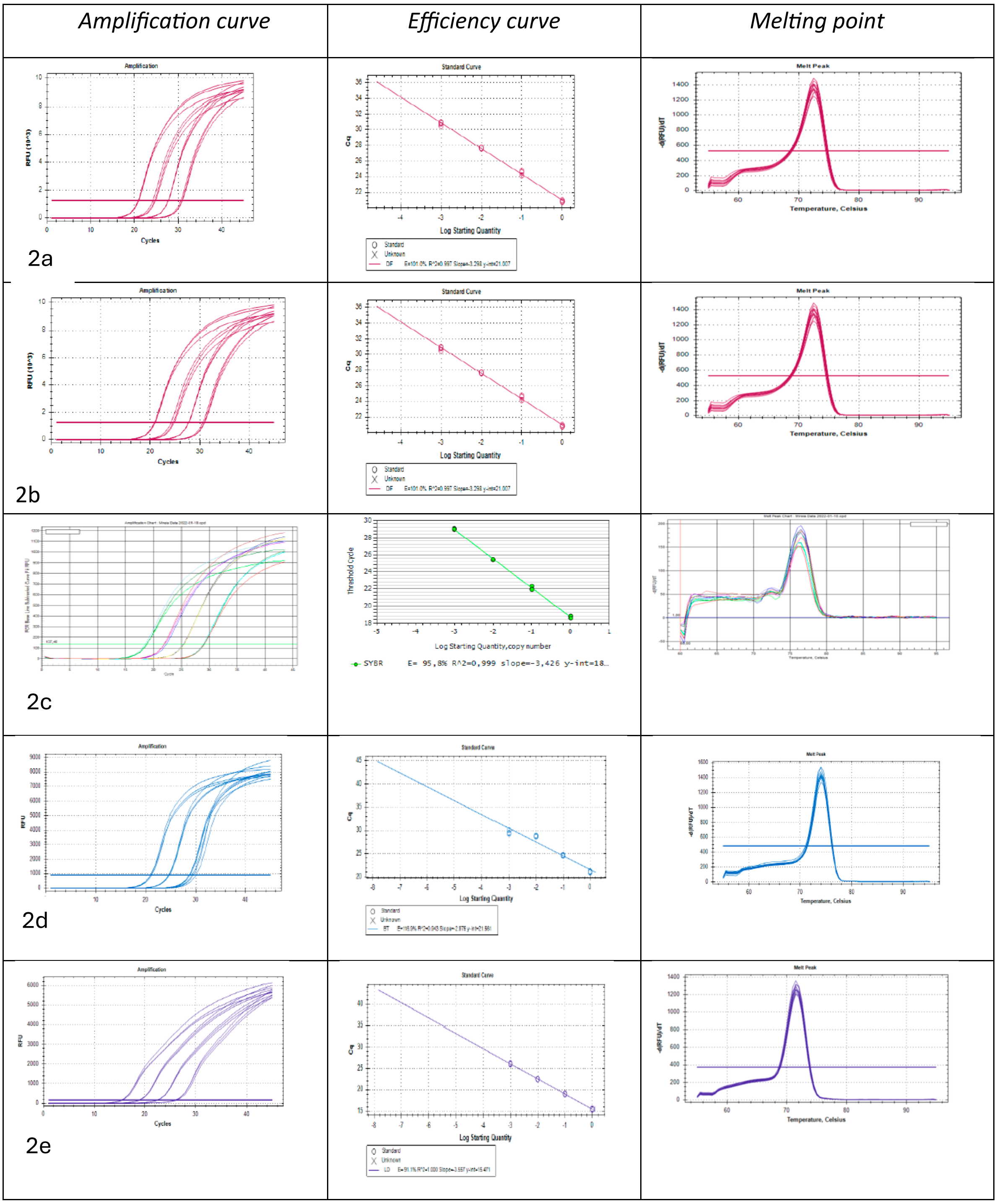

Figure S1: Amplification curves (left), efficiency curves (centre) and melting points (right): *D. pteronyssinus* (DPT: 2a*); D. farinae (*DF: 2b*); T. putrescentiae* (TP: 2c); *B. tropicalis* (BT: 2d); and *L. destructor* (LD: 2e).
