# Supplementary material for: Utility of the Ribosomal Gene 18S rRNA in the Classification of the Main House Dust Mites Involved in Hypersensitivity
Source: Int J Mol Sci. 2025 Oct 23;26(21):10308. doi: 10.3390/ijms262110308 (PMC12607703; doi:10.3390/ijms262110308)
Supplement: Supplementary file 1 [file ijms-26-10308-s001.zip › ijms-3768634-Figure S2.pdf]

Figura S2

Amplification curve	*Melt peak*
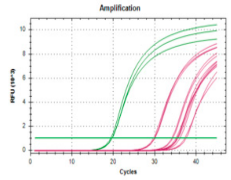 3a	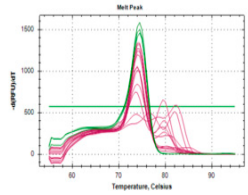 3b
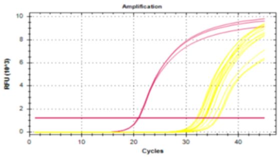 3c	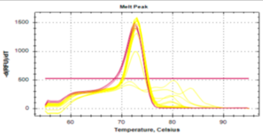 3d
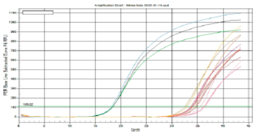 3e	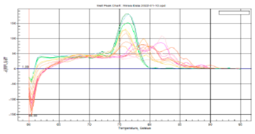 3f
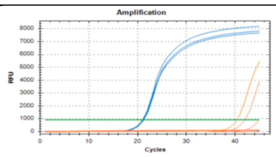 3g	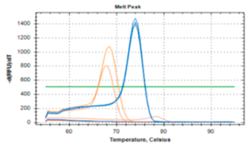 3h
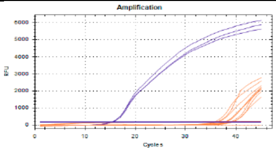 3i	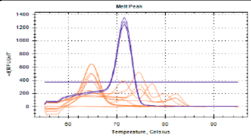 3j

Figure S2: Amplification curves (left) and melting points (right): *D. pteronyssinus* (3a,3b); *D. farinae* (3c, 3d); *T. putrescentiae* (3e,3f); *B. tropicalis* (3g,3h); and *L. destructor* (3i,3j).
