# Supplementary material for: Utility of the Ribosomal Gene 18S rRNA in the Classification of the Main House Dust Mites Involved in Hypersensitivity
Source: Int J Mol Sci. 2025 Oct 23;26(21):10308. doi: 10.3390/ijms262110308 (PMC12607703; doi:10.3390/ijms262110308)
Supplement: Supplementary file 1 [file ijms-26-10308-s001.zip › ijms-3768634-Figure S3.pdf]

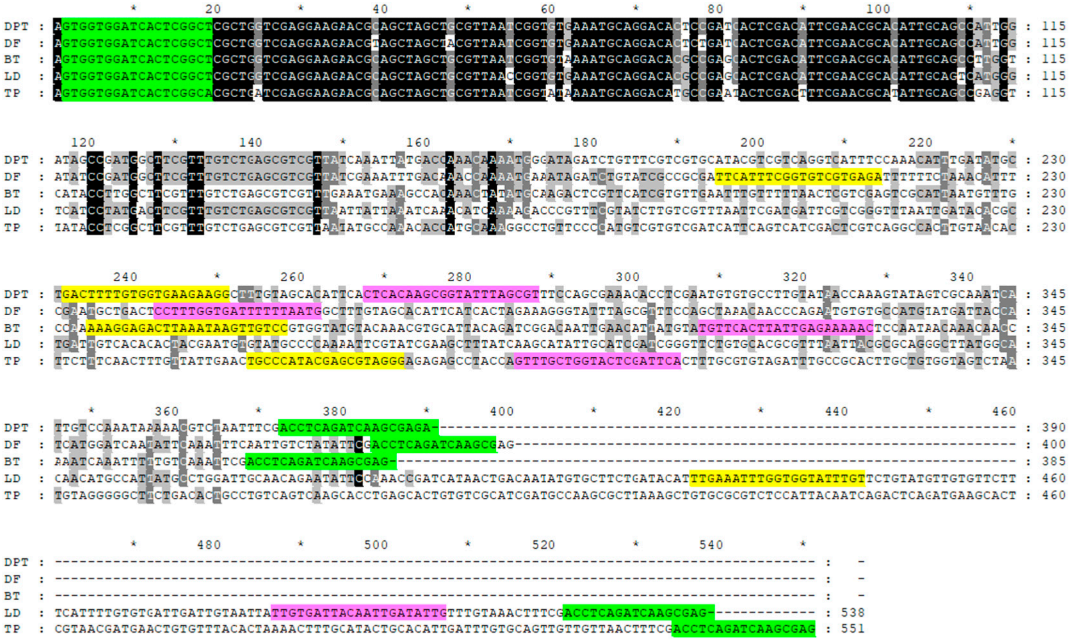

Figure S3: Alignment of the 5 species (Forward and Reverse. Green: barcodes; Yellow: forward of each species and Purple: reverse of each specie).
